# Forgotten Foreign Body Aspiration Presenting as Allergic Bronchopulmonary Aspergillosis: A Case Report

**DOI:** 10.7759/cureus.78280

**Published:** 2025-01-31

**Authors:** Nitesh Goyal, Soma Kiran, Dibakar Sahu, Sajal De

**Affiliations:** 1 Department of Pulmonary, Critical Care, and Sleep Medicine, All India Institute of Medical Sciences, Raipur, Raipur, IND

**Keywords:** airway foreign body, allergic bronchopulmonary aspergillosis, blood-stained sputum, flexible fiberoptic bronchoscopy (ffb), forgotten foreign body, high attenuation mucus, tracheobronchial tree

## Abstract

The usual presentation of foreign body (FB) aspiration into the tracheobronchial tree is a choking sensation followed by recurrent respiratory symptoms. Without a definitive history, the diagnosis of FB aspiration can be challenging. Here, we present a case of a 40-year-old male who presented with recurrent episodes of blood-stained sputum over the past six years. Contrast-enhanced computed tomography of the chest and serological investigations suggested allergic bronchopulmonary aspergillosis. Video bronchoscopy was performed for the hemoptysis workup, which identified and retrieved a plastic pen cap from the right lower lobe bronchus. On questioning, the patient disclosed a history of FB aspiration 20 years ago. Because he did not develop any immediate symptoms, he assumed that he probably swallowed the FB and never divulged it.

## Introduction

Foreign body aspiration (FB) in the tracheobronchial tree is common among children. The typical presentation is a choking event followed by chronic respiratory symptoms. The most common symptoms are cough, occurring in 58-96% of cases, followed by wheezing, dyspnea, hemoptysis, chest pain, and recurrent pneumonia [1]. The symptoms depend on various factors, including size, lodgment site, and degree of airway obstruction. The diagnosis is typically based on a history of aspiration, followed by confirmation by radiology or bronchoscopy. Diagnosing FB aspiration clinically without a history is challenging, especially in healthy adults. Bronchoscopy, especially rigid bronchoscopy, is commonly used to retrieve FB from the tracheobronchial tree.

## Case presentation

A man in his early 40s presented to our outpatient department with blood-stained sputum for the last 10 days. He had no other complaints or comorbidities. He was a never-smoker and a farmer by occupation. He had a past history of right-sided empyema seven years earlier, which was treated with intercostal tube drainage. He was clinicoradiologically diagnosed with pulmonary tuberculosis and received antitubercular treatment.

**Figure 1:**
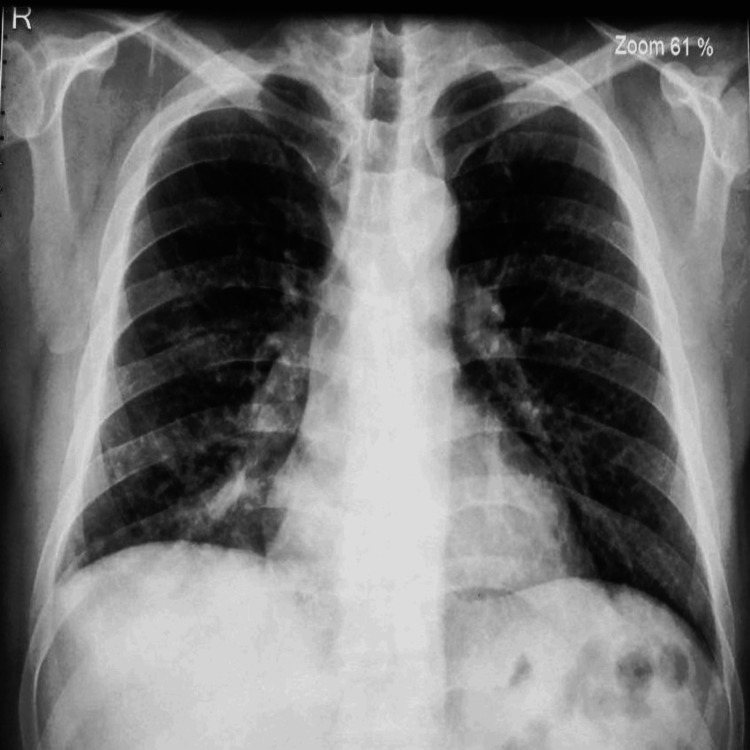
Chest X-ray, posteroanterior (PA) view.

**Figure 2:**
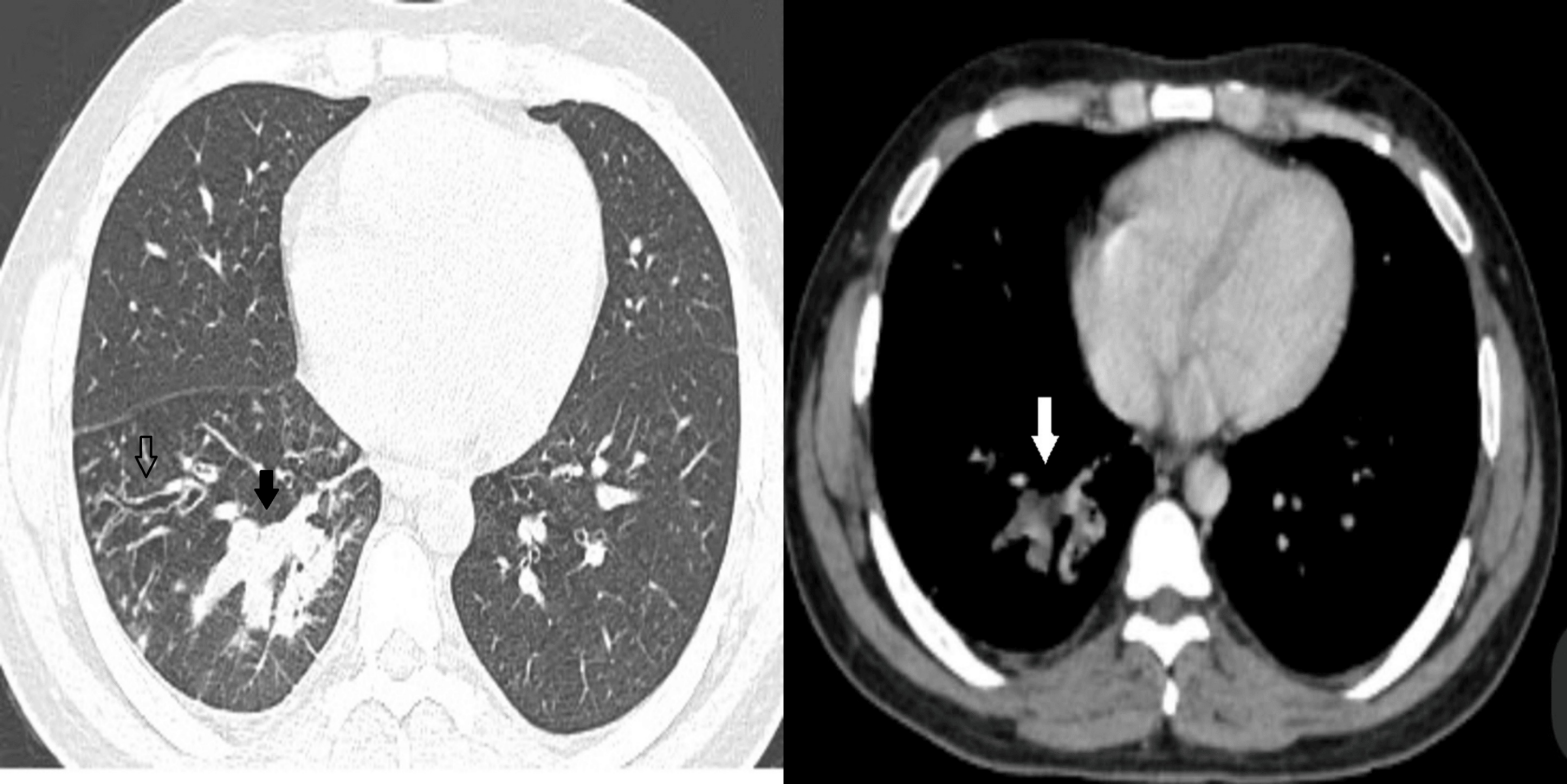
High-attenuation mucus (solid arrow) and bronchiectasis (open arrow) are seen in the axial image of the lung and mediastinal window of CECT of the thorax. CECT: contrast-enhanced computed tomography

His general physical and respiratory system examinations were unremarkable. His complete hemogram (hemoglobin: 13.9 g/dl; total leukocyte count: 8540/mm^3^, total eosinophil counts: 300/mm^3^) and other blood investigations, including the coagulation profile, were normal. *Mycobacterium* (Xpert MTB/RIF; Cepheid, Sunnyvale, California, United States), fungi, and pathogenic organisms were not isolated from his sputum. No apparent abnormalities were identified in his chest X-ray (Figure 1). Symptomatic treatment for hemoptysis was prescribed, and a contrast-enhanced computed tomography (CECT) of the thorax was advised. CECT demonstrated high-attenuation mucus (HAM) impaction with bronchiectasis in the right lower lobe (Figure 2). Based on the radiological features, he was investigated for allergic bronchopulmonary aspergillosis (ABPA). His serum total immunoglobulin E (1407 kU/L; normal: < 200 kU/L) and *Aspergillus fumigatus*-specific IgE (1.13 kU/L; normal: <0.35 kU/L) were elevated, and *A. fumigatus*-specific IgG (10.3 mgA/L; normal: <27mgA/L) was normal. His spirometry was normal (forced expiratory volume in one second (FEV_1_): 2.74 L (80% predicted); forced vital capacity (FVC): 3.42 L (82 % predicted); FEV_1_/FVC: 80.2%).

**Figure 3:**
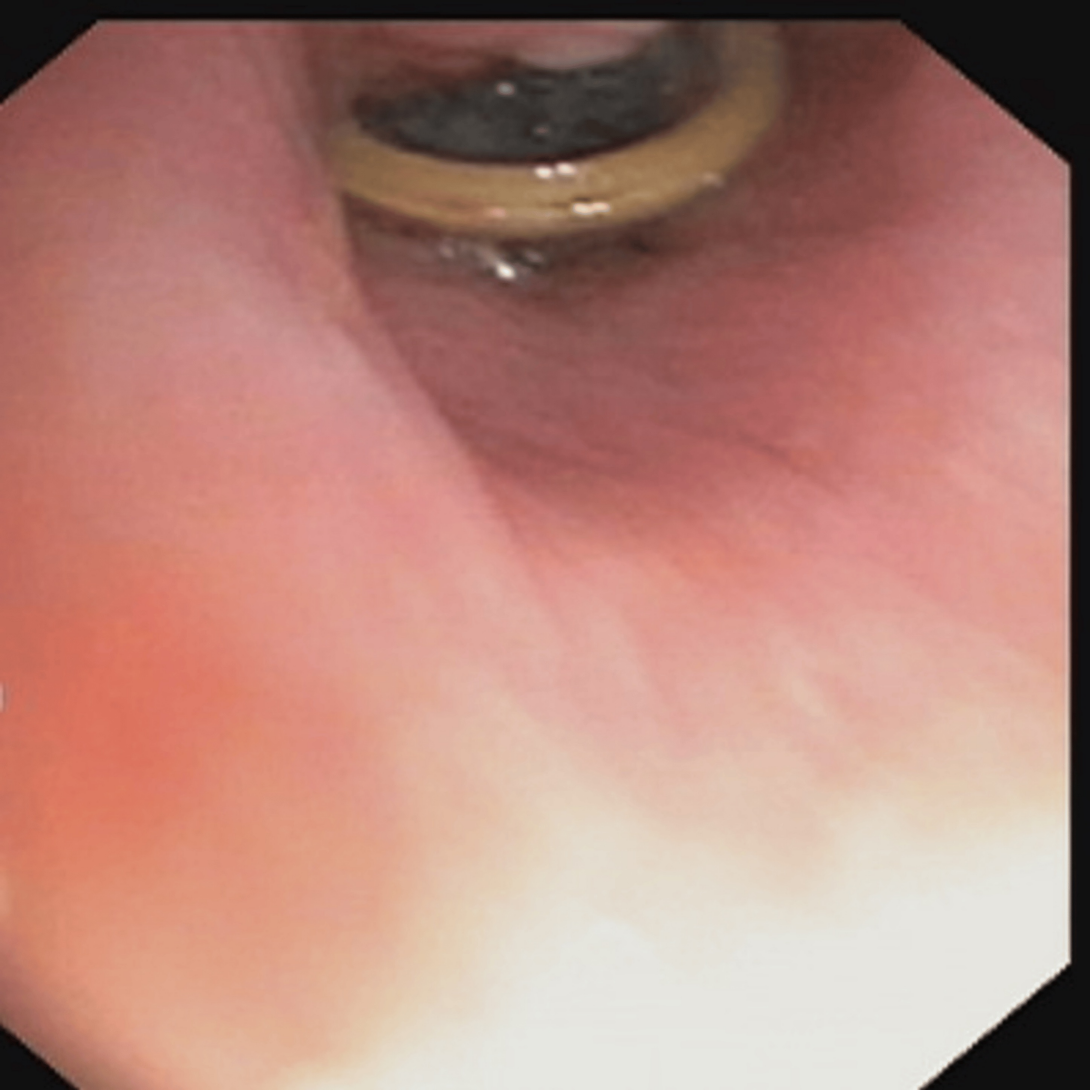
An impacted foreign body is seen in the bronchus during bronchoscopy.

**Figure 4:**
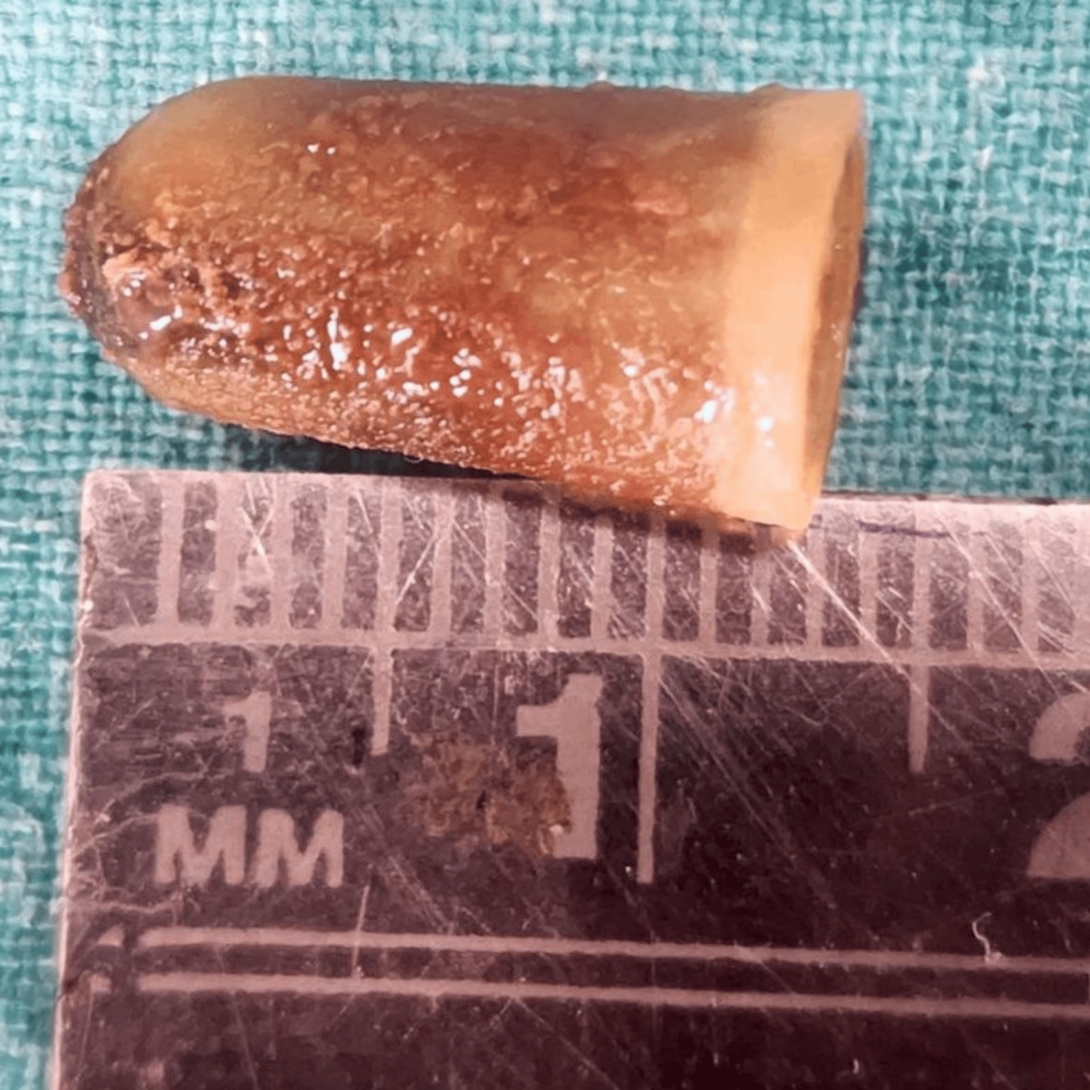
The yellow-colored cap of a pen retrieved from the bronchus

A provisional diagnosis of ABPA was made. A video bronchoscopy was performed to determine the etiology of the hemoptysis. During bronchoscopy, a yellow-colored, round FB was visualized in the right lower lobe bronchus just distal to the opening of the superior segment (Figure 3). The FB was successfully retrieved via the video bronchoscope using rotatable raw-tooth forceps with alligator jaws. The FB was a 15-mm-long plastic cap of a pen (Figure 4). On further questioning, the patient recalled a history of accidental aspiration of a pen cap while playing at 20 years of age. After aspiration, he had no immediate symptoms such as a choking sensation or cough and thought that he had swallowed the FB, which would be excreted through the stool. He did not recognize this as a significant event and never revealed it.

**Figure 5:**
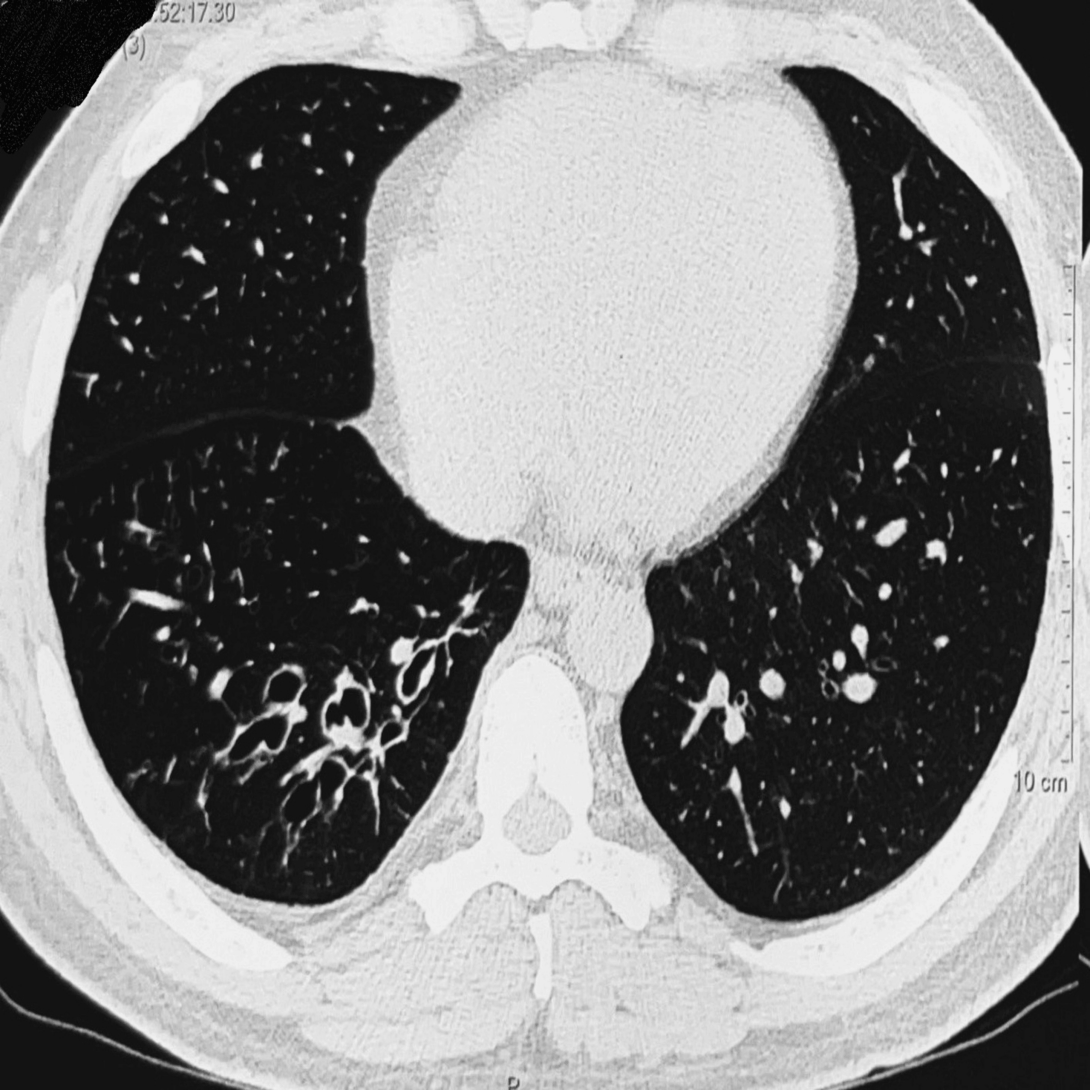
Lung window of follow-up CT scan three months after foreign body retrieval showing bronchiectasis in the right lower lobe.

Finally, we diagnosed the present case as a forgotten FB in the right lower lobe bronchus presented as ABPA. After FB removal, he was not given any treatment for the ABPA. He was reevaluated after three months and was found to be asymptomatic; however, his *A. fumigatus*-specific IgE remained elevated (1.95 kU/L), and the CECT of the chest showed persistence of bronchiectasis in the right lower lobe (Figure 5).

## Discussion

The natural presentation of FB aspiration is the sudden onset of vigorous cough or choking, followed by a variable asymptomatic period. The symptoms of FB aspiration depend on the degree of airway obstruction, duration, and location of the lodgment. It's not uncommon to forget about a history of FB aspiration, especially if the patient has no immediate symptoms. The most frequent presentations of late-diagnosed FB aspiration are consolidation and atelectasis of the lung (86.6%) [2].

Chest radiography can be challenging in detecting radiolucent FB. As the FB in our case was radiolucent, it was not identified in the chest X-ray and CECT thorax. The common radiological features of FB aspiration are airway obstruction, including collapse, bronchiectasis, and air trapping. Mucoid impaction due to plastic bronchitis can also mimic FB [3]. Bronchoscopy confirms the diagnosis of FB aspiration and is also used for retrieval. The extraction of long-standing FB using video bronchoscopic is challenging because of the surrounding granulation tissue. The amount of granulation tissue surrounding an FB differs between organic and inorganic FB [2]. Because of the inorganic nature of the current FB, the inflammatory response was minimal, and the airway was not completely obstructed.

ABPA may present as HAM on CT scans in up to 18% of cases [4]. The essential diagnostic criteria for ABPA, *A. fumigatus*-specific IgE ≥0.35 kUA/L and serum total IgE ≥500 IU/mL, were present in our patient [5]. However, he had no predisposing disease for ABPA, and his spirometry results were also normal. His *Aspergillus*-specific IgG was normal, and his total eosinophil count was <500 cells/µL. ABPA can be diagnosed by the presence of HAM on chest CT, even if a few other components of the diagnostic criteria are missing [4]. Bronchoscopy to collect respiratory samples for fungal culture is not routinely recommended for ABPA [5]. Bronchoscopy of ABPA is recommended in exceptional circumstances, such as unexplained hemoptysis, as in our case. The recent guidelines do not routinely recommend treating asymptomatic ABPA [5]. As the patient was asymptomatic, he was not offered ABPA-specific treatment. We believe that aspiration of FB led to obstruction, mucous plugging, and subsequent colonization of *A. fumigatus*.

## Conclusions

We reported a case of FB aspiration into the tracheobronchial tree in an adult who forgot about the previous history of aspiration, remained asymptomatic for years, and was diagnosed with ABPA based on radiological and serological parameters. The presence of localized bronchiectasis should be investigated for endoluminal obstruction by bronchoscopy.
